# In Situ Probing of CO_2_ Reduction on Cu‐Phthalocyanine‐Derived Cu_x_O Complex

**DOI:** 10.1002/advs.202304735

**Published:** 2023-11-29

**Authors:** Yongchan Jeong, Yongman Kim, Young Jae Kim, Jeong Young Park

**Affiliations:** ^1^ Center for Nanomaterials and Chemical Reactions Institute for Basic Science (IBS) 55, Expo‐ro, Yuseong‐gu Daejeon 34126 Republic of Korea; ^2^ Department of Chemistry Korea Advanced Institute of Science and Technology (KAIST) 291 Daehak‐ro, Yuseong‐gu Daejeon 34141 Republic of Korea

**Keywords:** CO_2_ reduction, Cu_x_O complex, Cu phthalocyanine, electrochemical scanning tunneling microscopy

## Abstract

An in situ measurement of a CO_2_ reduction reaction (CO_2_RR) in Cu‐phthalocyanine (CuPC) molecules adsorbed on an Au(111) surface is performed using electrochemical scanning tunneling microscopy. One intriguing phenomenon monitored in situ during CO_2_RR is that a well‐ordered CuPC adlayer is formed into an unsuspected nanocluster via molecular restructuring. At an electrode potential of −0.7 V versus Ag/AgCl, the Au surface is covered mainly with the clusters, showing restructuring‐induced CO_2_RR catalytic activity. Using a measurement of X‐ray photoelectron spectroscopy, it is revealed that the nanocluster represents a Cu complex with its formation mechanism. This work provides an in situ observation of the restructuring of the electrocatalyst to understand the surface‐reactive correlations and suggests the CO_2_RR catalyst works at a relatively low potential using the CuPC‐derived Cu nanoclusters as active species.

## Graphical abstract

EC‐STM images with atomic structures in Cu‐phthalocyanine adlayer on Au(111) surface at CO_2_ reduction reaction (CO_2_RR)‐inactivated and ‐activated surface morphologies. When the surface is mostly covered with the Cu_x_O complex, this morphological phase shows enhanced CO_2_RR activity.

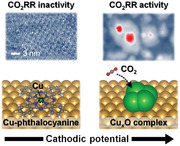

## 1 Introduction

Electrochemical CO_2_ reduction reaction (CO_2_RR) has been suggested as a significant alternative for mitigating the issues involved with CO_2_ gas emission and fossil fuel limits.^[^ ^1^ ^]^ CO_2_RR has distinct advantages, such as its mild operating conditions and the design of products by tuning reactions. Cu metal is the unique catalyst for CO_2_RR to produce value‐added hydrocarbons, because it allows the adsorption of CO intermediates.^[^ ^2^ ^]^ Moreover, depending on the exposed facet of Cu metal, the Cu metal can adjust the selectivity of CO_2_‐reduced products.^[^ ^2a,b,f,g^ ^]^ Cu‐based materials are also introduced as an alternative catalyst for CO_2_RR owing to the interesting performances of electrocatalytic reactions.^[^ ^3^ ^]^ Cu_2_O nanoparticles showed the facet‐dependent selectivity in the CO_2_RR for producing alcohols.^[^ ^3d,f,h^ ^]^ In addition, O‐derived Cu exhibited that it has remarkable CO_2_RR activity for C_2+_ products at relatively low overpotentials.^[^ ^3a–c,e^ ^]^

A fundamental understanding of the CO_2_RR mechanism is required in realistic reaction conditions to design ideal electrocatalysts for CO_2_RR. Since electrochemical scanning tunneling microscopy (EC‐STM) enables the investigation of morphological changes according to potential at the sub‐molecular level, EC‐STM studies on electrocatalytic reactions have been actively reported.^[^ ^4^ ^]^ Kim et al. evaluated a polycrystalline Cu electrode at the CO_2_RR‐induced potential using EC‐STM, conforming that the facet of the Cu electrode varies with the time exposed in 0.1 m KOH electrolytes.^[^ ^4a^ ^]^ Metal‐phthalocyanine (*Me*PC), which is one of the candidate catalysts due to its Metal‐N_4_ coordination, was investigated using EC‐STM, and the results showed that the shape contrast change related to the catalytic activity on *Me*PC is detected.^[^ ^4b,e^ ^]^ Kim et al. also carried out the EC‐STM study to investigate the adlayer of Mn porphyrin (MnP) on the Au(111) surface during an oxygen evolution reaction (OER).^[^ ^4g^ ^]^ They revealed the contribution of morphological phase transformation of MnP molecules to the catalytic activity of OER. The EC‐STM measurements facilitated the in situ approach in studying and understanding electrochemical reactions at the sub‐molecular level.^[^ ^5^ ^]^

The Cu‐based catalysts have led to various performances for CO_2_RR activity relying on components, structures, fabrication methods, etc. Recently, the reports introduced that metal oxides can be fabricated using *Me*PC and the fabricated metal oxides resulted in an improvement in electrochemical performance.^[^ ^6^ ^]^ In addition, the bimetallic materials can be suggested as an acceptable catalyst for the efficient electrochemical CO_2_RR due to adsorbed CO species and CO‐related intermediates toward multi‐carbon products.^[^ ^2^, ^7^ ^]^ Au,^[^ ^8^ ^]^ Ag,^[^ ^9^ ^]^ and Zn^[^ ^10^ ^]^ have been reported as efficient catalysts for CO production. Accordingly, they can be the most promising candidates for enhanced spillover of CO intermediates.

Based on these, we have designed a catalytic system of bimetallic tandem platforms utilizing Cu‐centered molecules with an Au electrode. We have focused on the CO_2_RR activity in CuPC and carried out the EC‐STM study of a CuPC adlayer on the Au(111) surface to observe CO_2_RR activity at a sub‐molecular level. The CuPC adlayer prepared by an immersion method showed well‐ordered configurations in CO_2_‐saturated 0.1 m KHCO_3_ electrolyte. However, at an electrode potential (*E*) of −0.7 V versus Ag/AgCl, the Au surface was covered mostly with unsuspected clusters in an EC‐STM image, and the reduction peak appeared in the cyclic voltammetry (CV) measurements, implying that this morphological surface is a CO_2_RR‐activated phase. With X‐ray photoelectron spectroscopy (XPS) measurements, we carefully revealed the components and the formation mechanism of the Cu‐based nanoclusters observed in the EC‐STM measurements.

## 2 Results and Discussion

**Figure 1:**
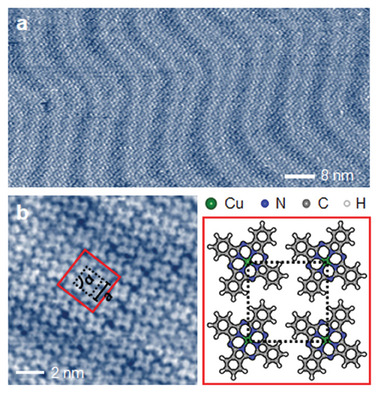
a) STM image of self‐assembled CuPC adlayer on Au(111) surface in CO_2_‐saturated 0.1 m KHCO_3_ measured at OCV with a tip bias voltage (*E*_tip_) of 200 mV, a tunneling current (*I*_tip_) of 0.45 nA, a Tip scan rate (*V*_tip_) of 912 nm s^−1^, and 512 × 512 pixels. b) High‐resolution STM image (*E*_tip_ = 200 mV, *I*_tip_ = 0.48 nA, *V*_tip_ = 343 nm s^−1^, and 512 × 512 pixels) and atomic configuration of CuPC adlayer.

We immersed a cleaned Au(111) sample in a benzene solution saturated by CuPC molecules to fabricate a CuPC adlayer. **Figure** **1**a shows the large‐scale STM image of the CuPC adlayer on the Au(111) surface in CO_2_‐saturated 0.1 m KHCO_3_ electrolyte at open circuit voltage (OCV). The adsorbed CuPC molecules exhibited a well‐ordered self‐assembly. To understand the detailed conformation from the CuPC adlayer, we peered into an enlarged STM image of the self‐assembled CuPC molecules, as shown in Figure 1b. Since a CuPC molecule consists of four isoindole groups linked by N atoms around the Cu atom, the STM image represented the CuPC molecule as a quatrefoil‐like shape with C_4_ symmetry. The four adsorbed CuPC molecules were placed at the corner of a rectangle‐like unit cell indicated by the black‐dotted box in Figure 1b. This unit cell showed lattice parameters of *a* = 14.6 ± 1 Å and angles of *α* = 90 ± 1^o^. These values are closely similar to the lattice parameters measured in *Me*PC molecules (*Me* = Fe, Co),^[^ ^4b,e^ ^]^ because the self‐assembly of *Me*PC molecules mainly occurs through the interactions between their isoindole groups.

**Figure 2:**
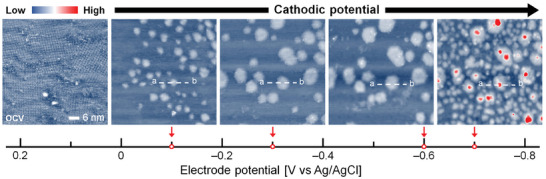
EC‐STM images of CuPC adlayer on Au(111) in CO_2_‐saturated 0.1 m KHCO_3_ electrolyte as a function of the applied electrode potential (*E*_tip_ = 200 mV and *I*_tip_ = 0.45 nA).

To investigate CO_2_RR on a CuPC molecule, we performed an EC‐STM measurement of the CuPC adlayer on the Au(111) surface (CuPC/Au(111) system) in a CO_2_‐saturated 0.1 m KHCO_3_ electrolyte. **Figure** **2** shows the EC‐STM images of the CuPC/Au(111) system as a function of the potential in the electrolyte. At a weak reduction wave (−0.1 V vs Ag/AgCl), we could not find the well‐ordered configuration of CuPC molecules, which is observed at OCV. However, we observed that nano‐sized clusters were newly emerged on the Au(111) surface. The emerged clusters had a size of ≈10 nm^2^ or less with a height of ≈0.22 nm (Figure S1, Supporting Information), and they were evenly distributed on the Au(111) surface. Since the clusters simultaneously appeared with no observation of well‐ordered CuPC molecules, we confirmed that the clusters disturbed the maintenance of the well‐ordered configuration of CuPC molecules. This is caused by inducing the cleavage of self‐assembled CuPC molecules by cluster formation, limiting the observation of the well‐ordered configurations. We changed the electrode potential from −0.1 to −0.3 V versus Ag/AgCl, showing that the area of the cluster became broader than the CuPC/Au(111) system at *E* = −0.1 V versus Ag/AgCl with no change in the height of the cluster. The area of clusters increased as more cathodic electrode potentials were applied. Due to the expanded cluster area, the coverage of total clusters also increased. On the other hand, the number of total clusters decreased (Figure S2, Supporting Information). From this, we estimated that the clusters manifested by applied cathodic potentials were not strongly fixed on the Au(111) surface and their area sizes were widened by merging with each other or growing. At *E* = −0.7 versus Ag/AgCl, the coverage of the total cluster considerably increased with the number of clusters, and the clusters that have a higher height than 0.22 nm were observed (Figure S1, Supporting Information). We confirmed the significant change of morphology at *E* = −0.7 versus Ag/AgCl.

To confirm the direct effect of Cu atom in CuPC on the formation of nanoclusters, we performed EC‐STM measurements using Ni‐phthalocyanine (NiPC) adlayer on the Au(111) surface. The NiPC adlayer also showed a well‐ordered configuration, and its configuration was maintained even when the cathodic potential was applied (Figure S3, Supporting Information) and turned to OCV again (Figure S4, Supporting Information). Furthermore, contrary to the CuPC/Au(111) system, the nano‐sized clusters did not appear in the NiPC/Au(111) system at a cathodic potential of −0.72 V versus Ag/AgCl (Figure S4c, Supporting Information). From this result, we verified the connection of the Cu atom in the CuPC molecule to the cluster formation observed in the CuPC/Au(111) system.

Ni atoms appear to be more strongly bound to the CO molecule than the Cu atom.^[^ ^11^ ^]^ Since the morphological change depended on the metal‐centered atom in *Me*PC, it is also necessary to investigate the contribution of CO_2_ gas, which provides CO molecules, to the cluster formation. We carried out the STM measurements after the electrochemical reaction on the CuPC/Au(111) system in an Ar‐saturated 0.1 m KHCO_3_ electrolyte. The STM image acquired after the electrochemical reaction (Figure S5b, Supporting Information) is quite similar to the one by the in situ method (Figure 2) in CO_2_‐saturated 0.1 m KHCO_3_ electrolyte, indicating the STM measurement after the reaction is in good agreement with the in situ EC‐STM results. We applied *E* = −0.7 V versus Ag/AgCl to CuPC/Au(111) system in Ar‐saturated 0.1 m KHCO_3_ electrolyte. Interestingly, the clusters did not appear, and a part of the well‐ordered configuration became disorderly (Figure S5c, Supporting Information). This means that CO_2_ gas‐provided CO molecules play an important role in cluster formation. From this result, we identified that the adsorption strength of the CO molecule on the metal atom affected the cluster formation. Furthermore, we estimated that the unstable CO─CuPC state induced by its weak CO─Cu bond enhanced the probability of separating Cu atom from CuPC.

**Figure 3:**
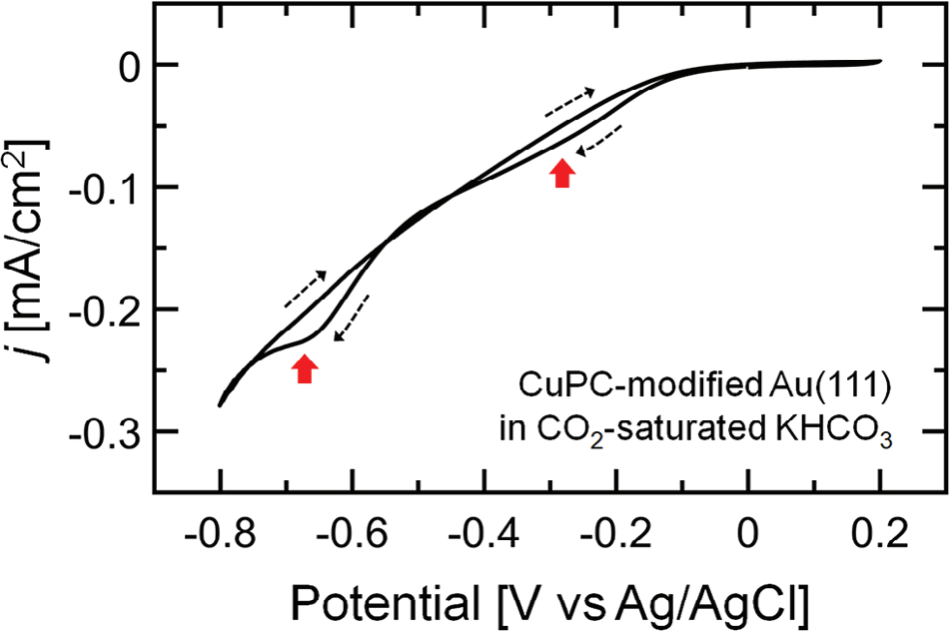
CV curve of CuPC‐modified Au(111) electrode in CO_2_‐saturate 0.1 m KHCO_3_ electrolyte. The black‐dotted arrow indicates the scan direction, and the scan rate is 0.03 V s^−1^. The red arrow means the reduction peaks.

We measured the CV of the CuPC‐modified Au(111) electrode in the CO_2_‐saturated 0.1 m KHCO_3_ electrolyte. The measured CV curve showed that the reduction peaks materialize near *E* = −0.2 and −0.7 V versus Ag/AgCl in the cathodic scan direction, as shown in the red arrows in **Figure** **3**. From this CV measurement, it was hard to reveal the contribution of the electrochemical reaction between CO_2_ gas and CuPC molecule to the reduction peak. Therefore, to confirm this contribution, we evaluated the dependence of CO_2_ gas and CuPC molecule on the reduction peak using the CV measurements of two cases: H_2_PC‐modified Au(111) electrode in CO_2_‐saturated 0.1 m KHCO_3_ electrolyte and CuPC‐modified Au(111) electrode in Ar‐saturated 0.1 m KHCO_3_ electrolyte. In the CV curves of these two cases (Figure S6, Supporting Information), we did not detect a reduction peak near *E* = −0.7 V versus Ag/AgCl. However, we observed a reduction peak near *E* = −0.2 V versus Ag/AgCl in the CO_2_‐saturated electrolyte (Figure S6a, Supporting Information). As a result, the reduction peaks observed in the CV curve of CuPC‐modified electrode in CO_2_‐saturated electrolyte (Figure 3) mainly originated in the CO_2_RR on CuPC molecules.

Thus, we successfully visualized the nucleation and growth behavior of cluster on CuPC/Au(111) tandem electrode for the first time, based on EC‐STM combined with CV measurements. At relatively low overpotentials, the progressive nucleation of CuPC into Cu‐based nanoclusters occurred during the electrochemical CO_2_ reduction. As the cathodic potential increased, each nucleus grew widely, and then finally merged with other nuclei. It is supported by the high coverage of clusters on the Au surface in the EC‐STM images and the observation of the reduction peak detected at *E* = −0.7 V versus Ag/AgCl. As a result, we can define that the cluster‐covered surface is the CO_2_RR‐activated morphology.

**Figure 4:**
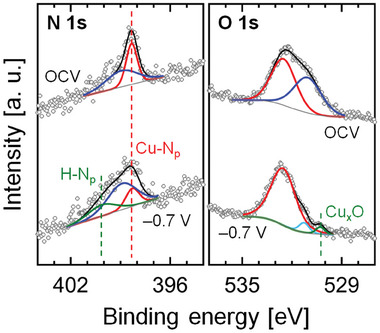
XPS spectra of N 1s and O 1s core level on CuPC‐modified Au(111) electrode. The electrodes were prepared before and after the application of cathodic electrode potential (−0.7 V versus Ag/AgCl) in a CO_2_‐saturated 0.1 m KHCO_3_ electrolyte.

We carried out the analysis of ex situ XPS to disclose the components of the surface morphology modified by the application of cathodic electrode potentials. The nano‐sized clusters, which are the main change of surface morphology at cathodic electrode potentials, were still identified in the EC‐STM image despite the electrode potential set to OCV (Figure S7, Supporting Information). Hence, the ex situ XPS measurement is available to investigate the surface morphological transition of CuPC/Au(111) system according to the application of electrode potential. **Figure** **4** shows the XPS spectra of N 1s and O 1s core levels for the CuPC‐modified Au(111) electrode, depending on the actively electrochemical reaction‐induced surface morphology. The N_p_ atom indicates the N atom of a pyrrolic ring in an isoindole group of CuPC molecule. Because the surface morphological phase for CO_2_RR activity was mainly identified near *E* = −0.7 V versus Ag/AgCl in the measured results, we prepared the two electrodes with the CO_2_RR‐inactivated and ‐activated surface morphologies (OCV and –0.7 V vs Ag/AgCl, respectively). In the CO_2_RR‐inactivated surface morphology, we identified a dominant peak in the N 1s region at 398.4 eV, which is assigned to the Cu─N_p_ bond in a CuPC molecule.^[^ ^12^ ^]^ In the CO_2_RR‐activated surface morphology, however, the intensity of the N 1s peak at ≈398.4 eV became weak. Moreover, the peak assigned to the H─N_p_ bond appeared at ≈400.1 eV despite no deposition of H_2_PC molecules in this system,^[^ ^13^ ^]^ and this peak closely resembled the one observed in the H_2_PC/Au(111) system (Figure S8, Supporting Information). This XPS result means that the high cathodic potential‐induced electrochemical reaction transforms some CuPC molecules into H_2_PC molecules or breaks down some CuPC molecules. We focused on the XPS measurements in the O 1s region. Interestingly, a weak intensity of peak was only detected at ≈530.3 eV, which is assigned to Cu_x_O,^[^ ^14^ ^]^ in the CO_2_RR‐activated surface morphology. To confirm this, we also investigate the XPS spectra of Cu 2p core level (Figure S9, Supporting Information). The CO_2_RR‐inactivated surface morphology mainly showed the peaks related to the CuPC molecule,^[^ ^12b^ ^]^ and the intensity of these peaks significantly decreased in the CO_2_RR‐activated surface morphology. Moreover, in the activated surface, we observed the peaks related to Cu_x_O.^[^ ^15^ ^]^ The key difference between CO_2_RR‐inactivated and ‐activated surface morphologies is the presence of nano‐sized clusters (Figure 2). As a result, the detection of peak assigned to Cu_x_O implied that the clusters observed in the EC‐STM measurements at cathodic electrode potentials represented Cu‐based electrocatalytic active complexes.

**Figure 5:**
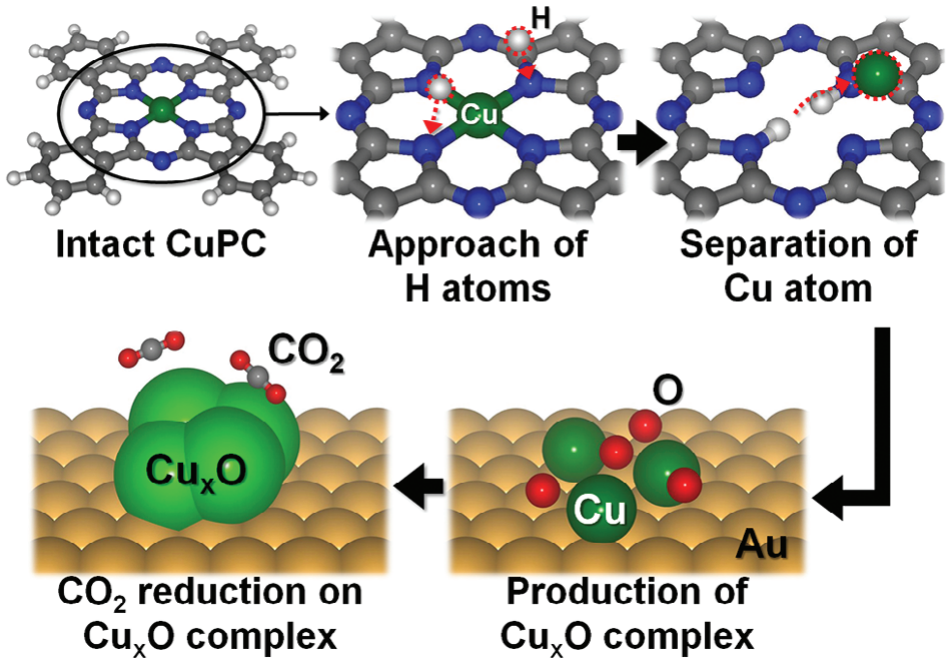
Perspective view of formation mechanism for the CuxO complex derived from CuPC molecule.

Our measurements provided the observation of the electrochemical reaction‐induced change in the CuPC/Au(111) system. Based on this, we can describe the CO_2_RR mechanism on the CuPC/Au(111) system, as shown in **Figure** **5**. We deduced the initial stage of cluster formation from the results of the N 1s (Figure 4) and Cu 2p XPS spectra (Figure S9, Supporting Information). Since H_2_PC molecules were confirmed despite the deposition of only CuPC molecules, we estimate that the H atoms kick out the Cu atom from a CuPC molecule, resulting in the substitution of H atoms to the Cu atom position. Due to this process, the Cu atom is separated from the CuPC molecule. In sequence, the separated Cu atom participates in the formation of a Cu_x_O complex. This is supported by the results of the O1s (Figure 4) and Cu 2p XPS spectra (Figure S9, Supporting Information). Through the collaboration between in situ EC‐STM measurement and ex situ XPS analysis, we revealed the origin of cluster formation. When the surface was almost covered with clusters in CuPC/Au(111) system, the reduction peak related to CO_2_RR was detected in the CV measurement (Figure 3). It exhibited that the Cu_x_O complex represented as the cluster in the EC‐STM images mainly contributed to CO_2_RR. Recently, the reports showed that the nanoparticles modified from the *Me*PC family enhance electrocatalytic properties due to appearance of active sites.^[^ ^16^ ^]^ From these reports, we hypothesize that these properties can mediate the tuning of the reduction potential for CO_2_RR in the CuPC‐derived Cu_x_O complex.

## 3 Conclusion

In conclusion, we demonstrated the overall process for CO_2_RR in CuPC/Au(111) system using the in situ and ex situ measurements. Using an in situ EC‐STM study, we have investigated the morphological phase for CO_2_RR in a well‐ordered CuPC adlayer on Au(111) surface. When the cathodic potentials were applied, the well‐ordered CuPC molecules were disassembled, but the unsuspected clusters manifested. At *E* = −0.7 V versus Ag/AgCl, we observed the cluster‐covered surface and the reduction peak for CO_2_RR in the EC‐STM and CV measurements, meaning that this surface is identified as the morphological phase for CO_2_RR activity. By supporting XPS measurements, we understood the formation mechanism of the cluster observed in the EC‐STM images. H atoms are substituted to the Cu atom position in the CuPC molecule, resulting in the separation of the Cu atom from the CuPC molecule. After this, the separated Cu atoms participated in the forming of the Cu_x_O complex scanned as the cluster in the EC‐STM images. Our study provides an in situ observation of morphological change according to electrochemical reaction in CuPC/Au(111) system and demonstrates a CO_2_RR activity on CuPC‐derived Cu_x_O complex.

## Conflict of Interest

The authors declare no conflict of interest.

## Supporting information

Supporting Information

Supplemental Movie 1

## Data Availability

The data that support the findings of this study are available from the corresponding author upon reasonable request.
