# Supplementary material for: In Situ Probing of CO2 Reduction on Cu‐Phthalocyanine‐Derived CuxO Complex
Source: Adv Sci (Weinh). 2023 Nov 29;11(4):2304735. doi: 10.1002/advs.202304735 (PMC10811478; doi:10.1002/advs.202304735)
Supplement: Supplementary file 1 — Supporting Information [file ADVS-11-2304735-s002.pdf]

ADVANCED SCIENCE
Open Access

Supporting Information

for *Adv. Sci.*, DOI 10.1002/advs.202304735

In Situ Probing of CO_2 Reduction on Cu-Phthalocyanine-Derived Cu_xO Complex

*Yongchan Jeong, Yongman Kim, Young Jae Kim and Jeong Young Park**

Supporting Information

In-Situ Probing of CO_2 Reduction on Cu-Phthalocyanine-Derived Cu_xO Complex

Yongchan Jeong, Yongman Kim, Young Jae Kim, and Jeong Young Park*

Experimental Procedures

Solution preparation: To fabricate a self-assembly of *Me*-phthalocyanine (*Me*PC, *Me* = Cu, Ni, H_2) molecules on Au(111) surface, we prepared the immersion solution by dissolving 0.1 mM *M*PC molecules (Sigma-Aldrich, CuPC: 252980, NiPC: 360635, and H_2PC: 253103) in benzene solution (Sigma-Aldrich, 270709). Potassium hydrogen carbonate (Sigma-Aldrich, $KHCO_3$: 237205) was dissolved in ultrapure water (>18.2 MΩ) to make an electrolyte solution.

Electrochemical scanning tunneling microscopy (EC-STM) measurements: All EC-STM experiments were performed in CO_2-saturated 0.1 M $KHCO_3$ electrolyte using RHK-STM (ATM300) and Autolab bipotentiostat (PGSTAT302N). We used the insulation material (Apiezon wax)-coated Pt-Ir tip in EC-STM measurement to minimize the influence of residual faradaic current. In the EC-STM cell, a working electrode was prepared by immersing the flame-annealed Au(111) electrode into the immersion solution. The prepared electrode obtained a highly-ordered CuPC adlayer on Au(111) surface. Pt wire was set up as reference and counter electrodes in the EC-STM cell.

Cyclic voltammetry (CV) measurements: We carried out CV measurements in a three-compartment electrochemical cell with N_2 atmosphere using Autolab potentiostat (PGSTAT302N). We prepared working electrodes in the same way as the case of EC-STM measurements. Ag/AgCl (3 M KCl) and Pt mesh were utilized as reference and counter electrodes, respectively.

X-ray photoelectron spectroscopy (XPS) measurements: Binding energies and elements were analyzed using XPS (Thermo VG Scientific, K-alpha) with Al K-alpha X-ray source (1486.7 eV). We prepared the Cu-modified Au(111) electrodes before and after the application of potential in CO_2-saturated 0.1 M $KHCO_3$ electrolyte. The prepared electrodes were loaded into XPS chamber while minimizing the contamination by air. The spectra presented in this work were calibrated against versus the Au $4f_{7/2}$ core level peak to 84.0 eV.

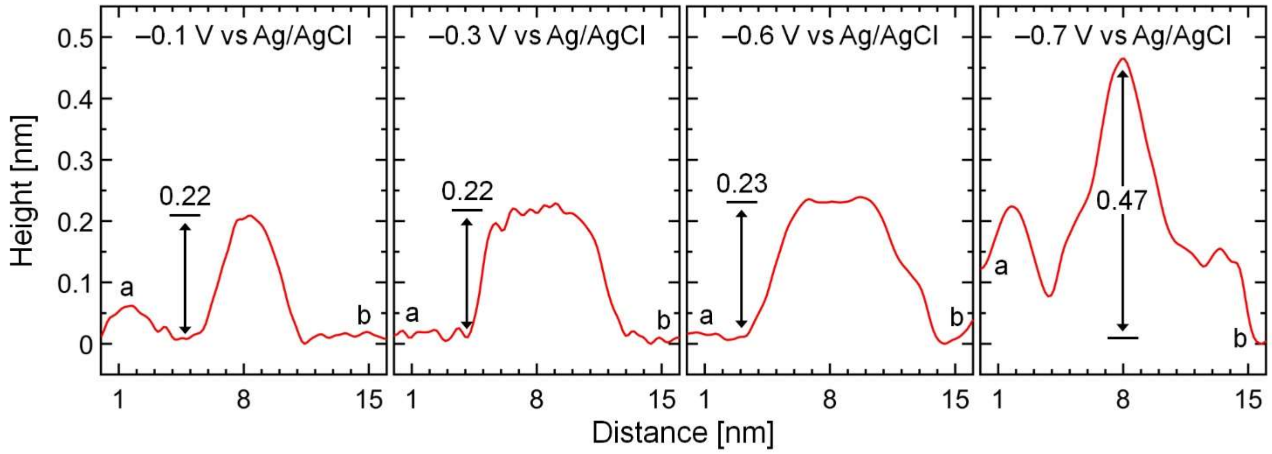

Figure S1. Cluster height profiles along the white-dotted line in Figure 2. At *E* = –0.7 V vs Ag/AgCl, the height of cluster becomes higher than that of *E* = –0.6 V vs Ag/AgCl.

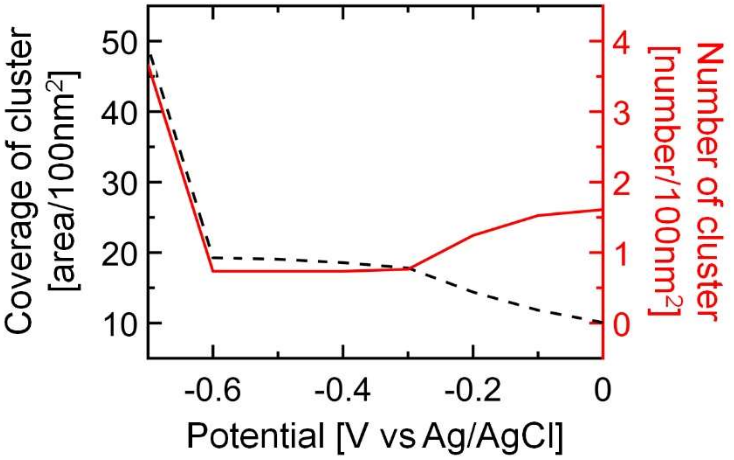

Figure S2. Coverage and number of cluster as a function of applied electrode potential. At *E* = –0.7 V vs Ag/AgCl, the coverage and number of clusters greatly increased.

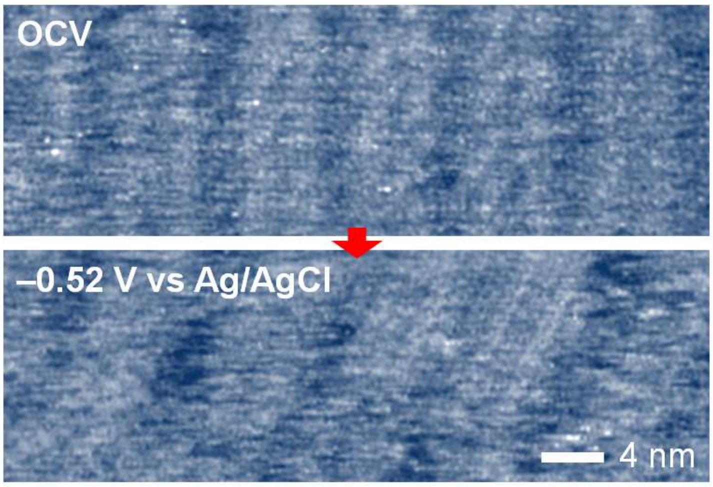

Figure S3. EC-STM images of NiPC/Au(111) system in CO_2-saturated 0.1 M $KHCO_3$ electrolyte at OCV and E = –0.52 V vs Ag/AgCl. Regardless of the application of electrode potential, the NiPC adlayer exhibited a well-ordered configuration.

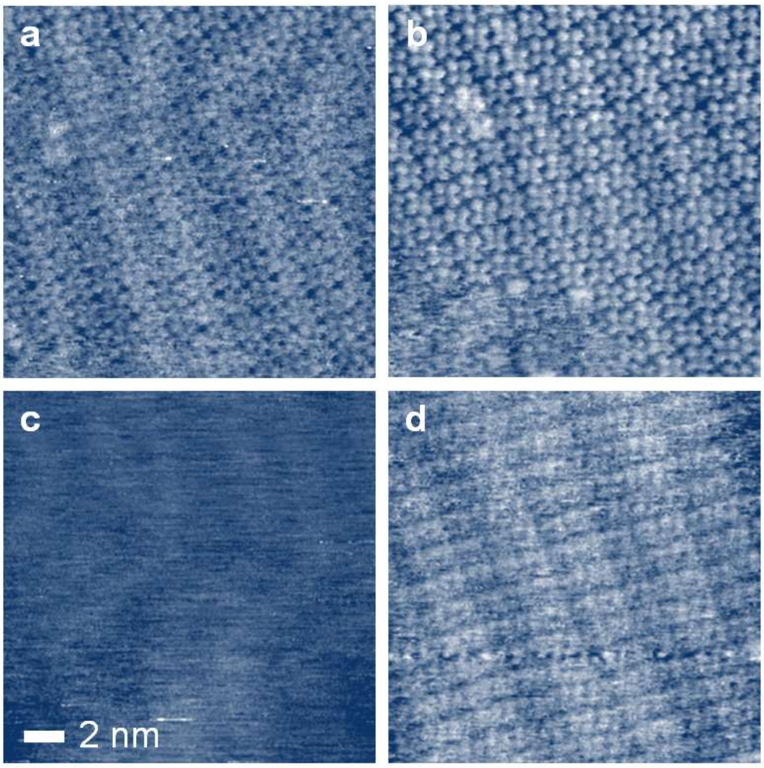

Figure S4. Sequential EC-STM images of NiPC/Au(111) system in CO_2-saturated 0.1 M $KHCO_3$ electrolyte at (a) OCV, (b) *E* = –0.12 V vs Ag/AgCl, (c) *E* = –0.72 V vs Ag/AgCl, and (d) OCV. The EC-STM image of (d) was obtained after scanning the one of (c). At *E* = –0.72 V vs Ag/AgCl, the well-ordered NiPC molecules disappeared. However, when the electode potential changed back to OCV, we again observed the well-ordered configuration.

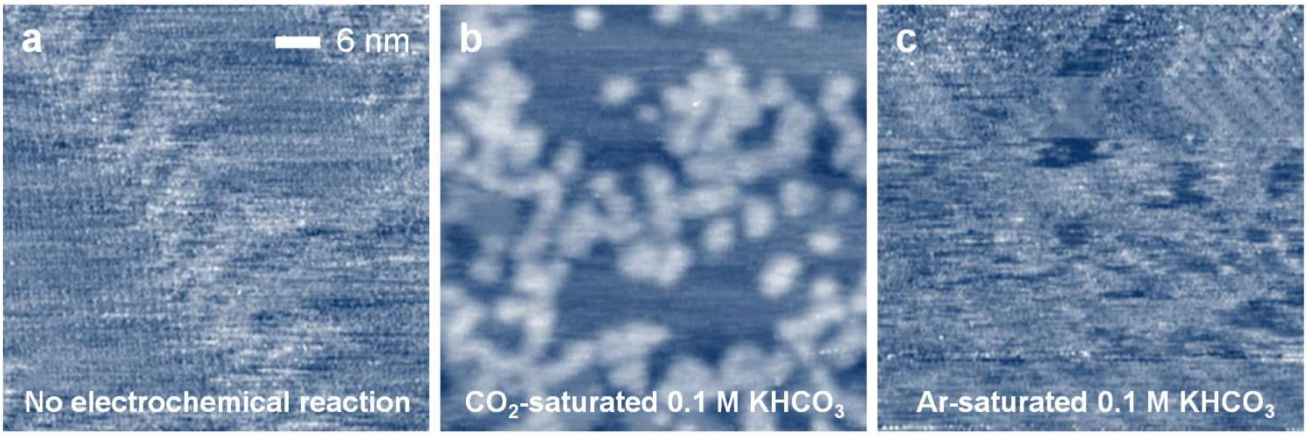

Figure S5. STM images of CuPC adlayer on Au(111) (a) before and (b, c) after electrochemical reaction. The STM images in Figure S5b and S5c were acquired by scanning the sample after the application of a potential of –0.7 V vs Ag/AgCl in CO_2- and Ar-saturated 0.1 M $KHCO_3$ electrolyte, respectively.

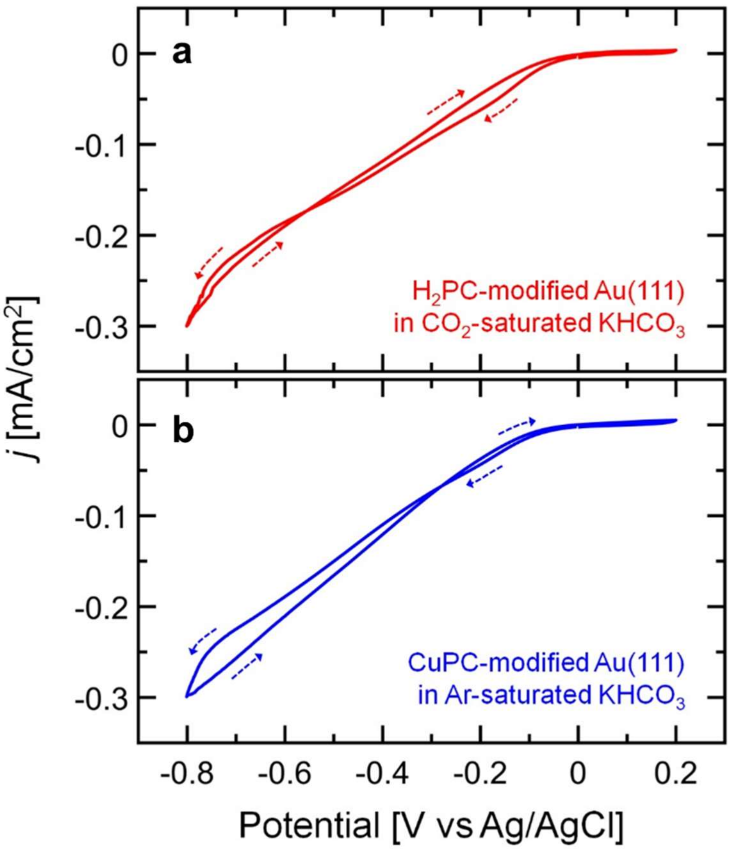

Figure S6. CV curves of H2PC-modified Au(111) electrode in CO_2-saturated 0.1 M $KHCO_3$ electrolyte and CuPC-modified Au(111) electrode in Ar-saturated 0.1 M $KHCO_3$ electrolyte. The dotted arrow indicates scan direction, and the scan rate is 0.03 V/s. In the measured CV results, we did not find a reduction peak.

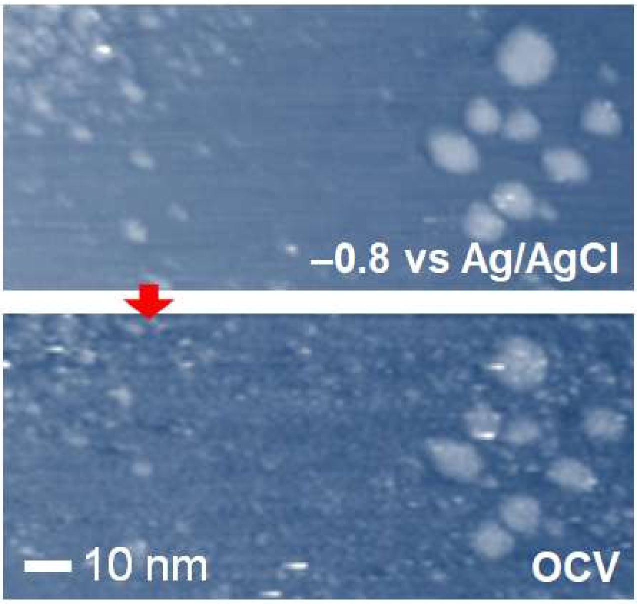

Figure S7. EC-STM images of CuPC/Au(111) system in CO_2-saturated 0.1 M $KHCO_3$ electrolyte at different electrode potentials. The EC-STM image at OCV was obtained after the application of an electrode potential of –0.8 V vs Ag/AgCl.

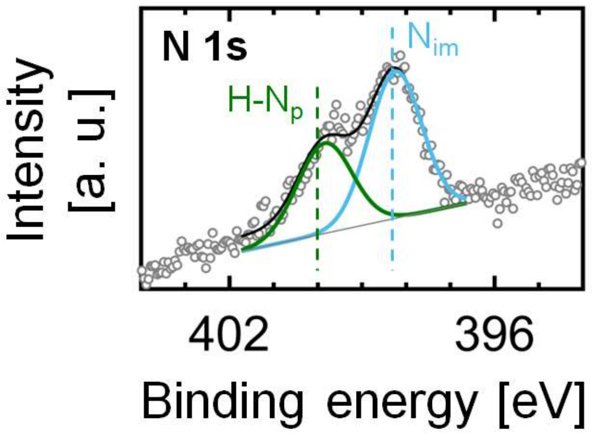

Figure S8. XPS spectrum of N 1s core level on H_2PC-modified Au(111) electrode. The N_{im} atom indicate the iminic N atom of pyrrolic ring in an isoindole group of H_2PC molecule.

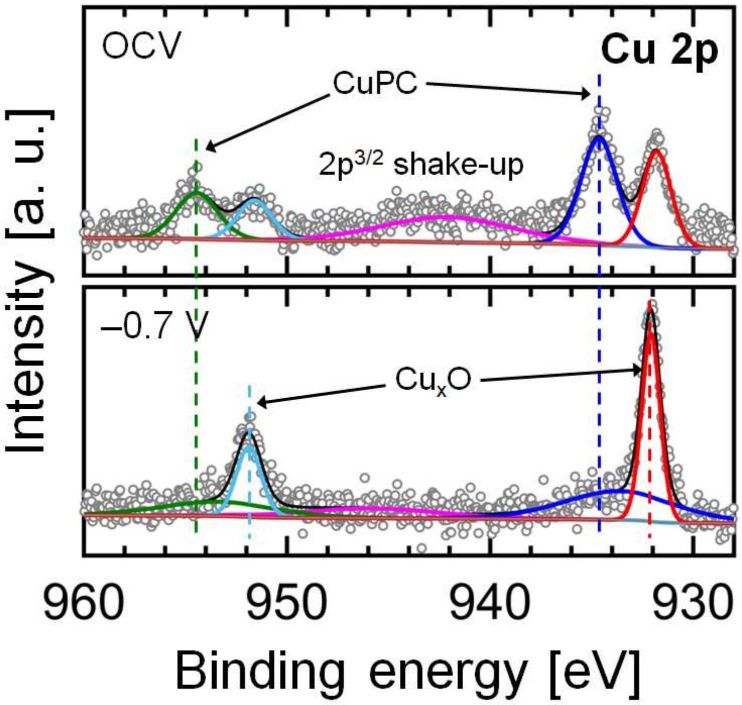

Figure S9. XPS spectra of Cu 2p core level on CuPC-modified Au(111) electrode. The electrodes were prepared before and after the application of cathodic electrode potential (–0.7 V vs. Ag/AgCl) in CO_2-saturated 0.1 M $KHCO_3$ electrolyte.
